# Procedural safeguards in remote criminal hearings

**DOI:** 10.3389/fpsyg.2026.1921601

**Published:** 2026-10-01

**Authors:** Ruibin Lu, Xiaohan Mei

**Affiliations:** 1 Criminal Justice Program, Stockton University, Galloway, NJ, United States; 2 School of Criminal Justice and Criminalistics, California State University, Los Angeles, Los Angeles, United States

**Keywords:** courtroom technology, procedural justice, remote criminal courts, statutory safeguard, video teleconferencing

## Abstract

**Introduction:**

Since the COVID-19 pandemic, criminal courts across the United States have started to conduct more remote proceedings using video teleconferencing technology. Although remote criminal hearings are often more cost-efficient and accessible than traditional in-person hearings, they introduce a set of new challenges to upholding defendants’ rights, such as the right to effective attorney representation and due process. The new hearing format also creates operational difficulties, such as technology malfunctions and the management of documents and exhibits.

**Methods:**

This study examines the procedural safeguards established in current state and federal laws and regulations for remote criminal proceedings.

**Result:**

A review of legal codes shows that, in most states, the statutory procedural safeguards are either inadequate or too superficial to address the challenges introduced by remote criminal proceedings.

**Discussion:**

The limitations identified in this study raise concerns about whether remote proceedings can fulfill equivalent standards as in-person proceedings. This study calls for legislators and court administrators to implement stronger procedural safeguards when authorizing remote criminal hearings.

## 1 Introduction

Remote criminal court hearings, conducted via video teleconferencing (VTC), have expanded substantially in the United States since the COVID-19 pandemic (Clarke and Smith, 2021; Jurva, 2021). Legislators have also cautiously embraced VTC, authorizing its use in pretrial hearings and, in some states, in evidentiary, trial, and/or sentencing hearings (Lu et al., 2026). The vast majority of the 50 states in the United States have permitted conducting at least some types of criminal pretrial proceedings remotely while more than half of the states permit using VTC in criminal trials and sentencing hearings, though most also impose restrictions such as with the defendant’s or both parties’ consent (Lu et al., 2026). This expansion is likely driven by the strength of remote hearings in saving costs and increasing court efficiency, especially by reducing the need for transporting detained defendants (Aggarwal et al., 2020; Clarke and Smith, 2021; Jones et al., 2023; Jurva, 2021; Turner, 2021). Additionally, VTC makes courts more accessible for individuals living in rural areas, with demanding schedules, or who are underprivileged because it reduces travel needs and is less disruptive to employment and childcare (Ariturk et al., 2020; National Center for State Courts [NCSC], 2025; Roby, 2024; Turner, 2021). The convenience of remote hearings has reduced failure-to-appear rates in some jurisdictions (Ariturk et al., 2020; National Center for State Courts [NCSC], 2025). Participating remotely may also be more desirable for victims and vulnerable witnesses who are concerned about facing the defendant or the emotional intensity of physical courtrooms (Fielding et al., 2020; McKay and Macintosh, 2024). Allowing public members to view court proceedings remotely increases the court’s transparency and boosts the public’s trust and understanding of the judicial system (Ariturk et al., 2020; Lederer, 2021; National Center for State Courts [NCSC], 2025).

But using VTC in criminal hearings also raises several constitutional concerns, including the right to due process, effective attorney representation, confrontation of witnesses, and a public trial (Benninger et al., 2021; Clarke and Smith, 2021; Diamond et al., 2010; Lederer, 2021; Poulin, 2004; Turner, 2021). It increases the difficulty of evaluating demeanor evidence because remote participants may behave and be interpreted differently than in a physical courtroom (Bandes and Feigenson, 2020; Benninger et al., 2021; Draper, 2024). Technical glitches and failures may also impair the parties’ ability to participate effectively or prejudice the defendant (Clarke and Smith, 2021; Nir and Musial, 2022; Turner, 2022). Although courts can mitigate these risks by obtaining defendants’ consent, they should also proactively implement protective measures that preserve the parties’ legal rights and address the negative impacts of VTC (Ariturk et al., 2020; European Commission for the Efficiency of Justice [CEPEJ], 2021; Poulin, 2004).

Many courts choose to keep the VTC option post-pandemic (Reed, 2023). In a survey of state courts in 2025, 80 percent of respondents indicated that their courts conducted or participated in virtual hearings (Thomson Reuters Institute and National Center for State Courts Ai Policy Consortium on Law and Courts, 2025). Therefore, it is important to evaluate the extent to which legislators and court administrators implement safeguards in remote criminal proceedings. Providing robust mandatory safeguards enhances procedural justice in remote hearings, which in turn increases the likelihood that defendants accept the outcomes of their hearings and strengthens public trust in the judicial system (Tyler, 1990; Tyler, 2003). For this purpose, we conducted a content analysis study to review the procedural safeguards adopted in state and federal legal codes in the United States. Specifically, we focus on five concerns, including (1) defendants’ right to effective representation and confidential attorney-client communication, (2) improper influence over remote witnesses, (3) audio and/or video quality, (4) remote document and exhibit management, and (5) public access to remote hearings. The findings of this study will contribute to conversations about whether criminal courts should expand or limit VTC use and help courts strengthen the integrity of remote hearings.

## 2 Concerns about remote criminal proceedings and recommended protections

### 2.1 Defendants’ right to effective representation and confidential attorney-client communication

Legal scholars and court professionals have raised several theoretical or perceived concerns for remote criminal hearings. First, remote hearings may infringe on defendants’ right to effective assistance of counsel because the quality of legal representation may diminish greatly when defendants and their attorneys appear from different locations (Ariturk et al., 2020). Although recent technology, such as the breakout room function, has made it easier for participants to converse privately, defense attorneys’ ability to have subtle, immediate conversations with their clients is still hindered (Benninger et al., 2021; Clarke and Smith, 2021; Roby, 2024). For example, a defendant may hesitate to use private communication channels for fear of disrupting the court (Valchev, 2020). Attorneys can no longer quietly nudge a defendant on what to say or withhold during a remote hearing, or stop a defendant from doing something such as swearing or interrupting the judge by kicking him/her under the table (Bannon and Keith, 2021; Benninger et al., 2021; Clarke and Smith, 2021). VTC may also hinder defense attorneys’ ability to build trust with their clients and gather information that may lead to more favorable outcomes before the hearings (Benninger et al., 2021; Clark, 2021; Diamond et al., 2010; Fielding et al., 2020). In addition, even when remote communication platforms offer private communication channels, the setup in detention facilities may undermine attorney-client confidentiality (Gibbs, 2017; Roby, 2024). Interviews with U.S. Magistrate Judges about federal detention hearings during the COVID-19 pandemic showed that guards could frequently overhear defendants’ conversations with their counsel because they were stationed in the same room (Roby, 2024).

To address these concerns, courts should ensure defendants have the right to privately communicate with their lawyers not only during, but also before remote proceedings (European Commission for the Efficiency of Justice [CEPEJ], 2021; Prosecutors’ Center for Excellence, 2024; National Center for State Courts [NCSC], 2025; Turner, 2022). These communications should occur over a secure, confidential system that is free from surveillance, interception, or other people eavesdropping in the vicinity (European Commission for the Efficiency of Justice [CEPEJ], 2021). The communication channel must be stable enough to prevent technical failures from disrupting attorney-client conversations (Valchev, 2020). Judges can further uphold the right to private attorney-client communication by informing defendants of this right, explaining how to use the private channels, and taking frequent breaks to facilitate confidential conversations (Roby, 2024; Rossner and Tait, 2024; Turner, 2021; Turner, 2022). Finally, having attorneys and defendants appear together from the same location for a remote hearing alleviates concerns about communication effectiveness (Clarke and Smith, 2021; Turner, 2022).

### 2.2 Improper influence over remote witnesses

Accepting remote testimony raises concerns about witness coaching and coercion. Surveys and interviews of judges and court professionals during the COVID-19 pandemic showed that many court actors were concerned about being unable to tell whether remote witnesses were coached, threatened, or secretly referring to a document off-camera (Benninger et al., 2021; Jurva, 2021; Roby, 2024). In 2021, a defendant in Michigan was caught discreetly threatening the victim during a remote hearing, pretending to be at a different location while hiding in the same room as the victim (Li, 2021). As remote criminal trials are permitted under certain circumstances in more than half of the 50 states, courts must implement measures to mitigate the risk of witness coaching and coercion, even when remote testimony is rarely used (Lu et al., 2026). Therefore, courts that authorize remote testimony must implement safeguards to prevent improper pressure or influence on the witness (European Commission for the Efficiency of Justice [CEPEJ], 2021). Suggestions include proactively inquiring whether a witness is assisted by another person or a document, requesting camera angles to reveal a witness’s surroundings, requesting witnesses to appear from court-approved locations where a court officer can be present, and dispatching a neutral monitor to the witness’s location (Benninger et al., 2021; Lederer, 2021; Prosecutors’ Center for Excellence, 2024). Courts should also keep the remote witnesses’ privacy in mind when adopting the preventive measures (Draper, 2024).

### 2.3 Operational challenges

Operational challenges such as technology malfunctions may violate the Due Process Clause if they obstruct a participant from observing, hearing, or participating in a material part of a remote criminal hearing (Turner, 2022). However, observations of remote proceedings revealed that technical issues occurred in a notable number of hearings, ranging from being occasionally unable to hear a participant, audio lag causing participants to talk over each other, and more serious problems causing a participant to be temporarily or permanently disconnected (Nir and Musial, 2022; Turner, 2022). Similarly, being unable to access hearing documents, evidence, images, or communicate with the court about these case materials may cause serious constitutional violations (Bannon and Keith, 2021; Jurva, 2021). Yet, document management was a common challenge when criminal hearings transitioned to the remote format during the early onset of COVID-19 (Bannon and Keith, 2021; Jurva, 2021; Roby, 2024).

The European Commission for the Efficiency of Justice (European Commission for the Efficiency of Justice [CEPEJ], 2021) asks courts to continuously monitor the quality of audio and video transmission in remote proceedings to ensure that all participants can see and hear each other. When technical challenges arise, judges can repeat the affected portions caused by brief and minor problems or suspend or reschedule the hearing if the issues are severe enough to threaten its fairness (European Commission for the Efficiency of Justice [CEPEJ], 2021; Turner, 2022). Courts can also prevent technical challenges from undermining the fairness of remote hearings by allowing participants to test the audio and video quality, performing regular equipment checks, informing defendants on how to report technical issues, and regularly checking that all participants can hear, see, and participate fully (European Commission for the Efficiency of Justice [CEPEJ], 2021; Fielding et al., 2020; National Center for State Courts [NCSC], 2025; Turner, 2022). In addition, courts must ensure all participants, regardless of their locations, can observe and/or hear all case materials presented during remote proceedings (European Commission for the Efficiency of Justice [CEPEJ], 2021). Courts can begin by outlining clear policies on acceptable ways to submit and present documents, exhibits, and other materials before and during a remote hearing (Bannon and Keith, 2021; European Commission for the Efficiency of Justice [CEPEJ], 2021). Courts may consider visually displaying documents, photos, and videos on computer screens and monitors, and giving parties opportunities to examine physical exhibits before remote proceedings (Prosecutors’ Center for Excellence, 2024).

### 2.4 Public access to remote hearings

Finally, to protect defendants’ right to a public trial and uphold judicial transparency, courts must provide means for public members to access remote hearings that are usually open to the public (European Commission for the Efficiency of Justice [CEPEJ], 2021; Prosecutors’ Center for Excellence, 2024; Turner, 2021). This may be achieved by livestreaming court proceedings, posting audiovisual recordings online, or broadcasting remote proceedings on monitors displayed inside courtrooms (European Commission for the Efficiency of Justice [CEPEJ], 2021; Prosecutors’ Center for Excellence, 2024; National Center for State Courts [NCSC], 2025; Turner, 2021). However, courts should also protect the parties’ privacy and ensure that sensitive or private information, such as documents containing personal identifiers, is not inadvertently broadcast (European Commission for the Efficiency of Justice [CEPEJ], 2021; Prosecutors’ Center for Excellence, 2024; Turner, 2021). National Center for State Courts [NCSC] (2022) and European Commission for the Efficiency of Justice [CEPEJ] (2021) recommend methods such as prohibiting unauthorized photographing, recording, or other forms of dissemination, creating public viewing rooms in the courthouse, or streaming remote hearings with a delayed signal. Boulanger-Bonnelly (2022) also recommends requiring public members who wish to observe remote hearings to complete a registration form providing basic personal information, both to inform observers of the rules governing public access and to discourage inappropriate behavior or misuse of the anonymity afforded by remote observation.

To mitigate these concerns, legislators and court administrators should develop a legal framework that establishes procedural safeguards for conducting remote criminal proceedings (Beazley and Van de Gaer, 2024; European Commission for the Efficiency of Justice [CEPEJ], 2021). Although there is currently limited and conflicting evidence on how remote communication affects criminal case processing and outcomes (Diamond et al., 2010; Fielding et al., 2020; Harris, 2023; Kim, 2021; Roby, 2024), the theoretical and perceived concerns warrant robust safeguards to minimize the potential risks. Codifying safeguards into state and federal laws would promote courts’ fairness and transparency, encourage policymakers to anticipate and prepare for challenges, set clear and consistent standards, and motivate judges to proactively protect the parties’ procedural rights (European Commission for the Efficiency of Justice [CEPEJ], 2021; National Center for State Courts [NCSC], 2025; Turner, 2022).

## 3 Methodology

In the current study, we performed a content analysis that examines the common themes of existing operational standards for remote criminal proceedings as codified in state and federal laws. The purpose of this study is to assess how the U.S. judicial system protects due process and addresses the concerns of remote proceedings. However, it does not assess the constitutionality of a particular doctrine or whether a state’s law fulfills all procedural justice standards. Instead, this study reviews the extent to which legislators and court administrators anticipated and expressly addressed challenges that may arise in remote criminal proceedings, including (1) barriers to defendants’ right to effective assistance of counsel, (2) improper influence over remote witnesses, (3) poor VTC audio and/or video quality, (4) ineffective document and exhibit management, and (5) public access to remote hearings.

We searched for state and federal statutes, court rules, and administrative orders that are current as of September 2025 that govern criminal trial and pre-trial proceedings in which a court participant appears remotely.^1^ The search was conducted using Westlaw and the websites of all fifty states’ judicial branches. We investigated statutory and regulatory protections in proceedings in which at least one participant is permitted to appear remotely using technologies such as interactive audiovisual systems, video conferencing, telephone, and closed-circuit television. One team member searched the Westlaw database using both Boolean searches and targeted searches within statutes and court rules likely to contain provisions governing remote criminal hearings and remote appearances. A second team member searched the official websites of all fifty state judiciaries to identify additional administrative orders and directives and supplemented these searches with general Google searches to identify materials that might not have been readily accessible through state court websites. Using content analysis, one researcher examined each state’s legal codes to identify whether and how states regulate (1) confidential attorney-client communication; (2) simultaneous audio and video communication; (3) public access to remote proceedings; (4) access to documents and physical evidence; and (5) safeguards for remote testimony. To reduce the risk of single-coder bias, the researcher used a standardized coding framework and maintained a research journal documenting coding definitions, procedures, and analytic decisions. The other team member reviewed the statutory evidence supporting the coding decisions to help mitigate potential subjectivity.

The analysis operates within the following parameters. First, the study is restricted to statutes and regulations that apply to all trial courts in a state. It excludes local court rules or administrative orders, pilot programs, regulations governing appellate-level review, and emergency orders related to COVID-19 or other public health crises. Second, this study focuses on proceedings in which a defendant’s in-person and/or remote presence is required. However, the analysis does not differentiate between the significance of various proceedings and instead treats the protections offered in all evidentiary and non-evidentiary hearings equally. Additionally, it does not examine whether the remote appearance is conditioned on factors such as whether a defendant is detained or has consent to appear remotely. The study also does not distinguish between fully remote hearings, where all participants appear remotely, and hybrid hearings, where some participants appear via VTC while others appear in the courtroom. Third, the study excludes regulations that specifically govern the appearance of special populations, including child victims, military personnel, or victims of human trafficking or sexual assault. These parameters may slightly limit the study’s ability to analyze certain procedural protections. However, the effect is expected to be minimal. These parameters are adopted to ensure the analysis reflects the norms of each state’s practice and account for a broad range of safeguards used in different types of proceedings. A detailed summary of the protections offered by each state is available in Appendix 1.

## 4 Findings

### 4.1 Defendants’ right to effective representation and confidential attorney-client communication

As shown in Table 1 and Figure 1, 34 states and the District of Columbia (70%) provide some form of regulatory protection for defendants’ ability to communicate privately with counsel during remote criminal hearings. In most of these states, the law requires courts to provide private and/or confidential attorney-client communication, particularly when defendants and counsel participate from different physical locations. For example, when VTC technology is used in remote criminal hearings in Wisconsin, “if not in each other’s physical presence, a separate private voice communication facility shall be available so that the defendant or respondent and his or her attorney are able to communicate privately during the entire proceeding” [Wis. Stat. Ann. §885.54 (2008)]. Arizona, Colorado, and Ohio further require courts to inform defendants of the available methods for confidential communication. According to Ohio Rules of Criminal Procedure, Rule 43 (2023), a condition for allowing defendants to appear remotely is that “the court makes provision to allow for private communication between the defendant and counsel. The court shall inform the defendant on the record how to, at any time, communicate privately with counsel.” Six states and the District of Columbia extend this protection to both before and during a criminal hearing. For example, California Penal Code §977 (2024) states that private communication must be available when defense attorneys and defendants are at different physical locations prior to and during a proceeding that uses remote technology.

**TABLE 1:** Statutes and regulations on protecting confidential attorney-client communication in remote criminal hearings.

Protected (*N* = 35; 70%)	No regulation (*N* = 15; 30%)
Alabama Alaska Arizona (*before and during a hearing; inform defendants*) Arkansas California (*before and during a hearing*) Colorado (*inform defendants*) Connecticut (*before and during a hearing*) District of Columbia (*before and during a hearing*) Georgia Idaho Illinois Kansas Louisiana (*before and during a hearing*) Maryland Michigan Minnesota Mississippi (*before and during a hearing*) Montana Nebraska Nevada New Mexico New York North Carolina North Dakota Ohio (*inform defendants*) Oklahoma Oregon Pennsylvania (*before and during a hearing*) South Carolina South Dakota Texas Vermont Washington Wisconsin Wyoming	Delaware Federal Florida Hawaii Iowa Maine Massachusetts Missouri New Hampshire New Jersey Rhode Island Tennessee Utah Virginia West Virginia

**FIGURE 1:**
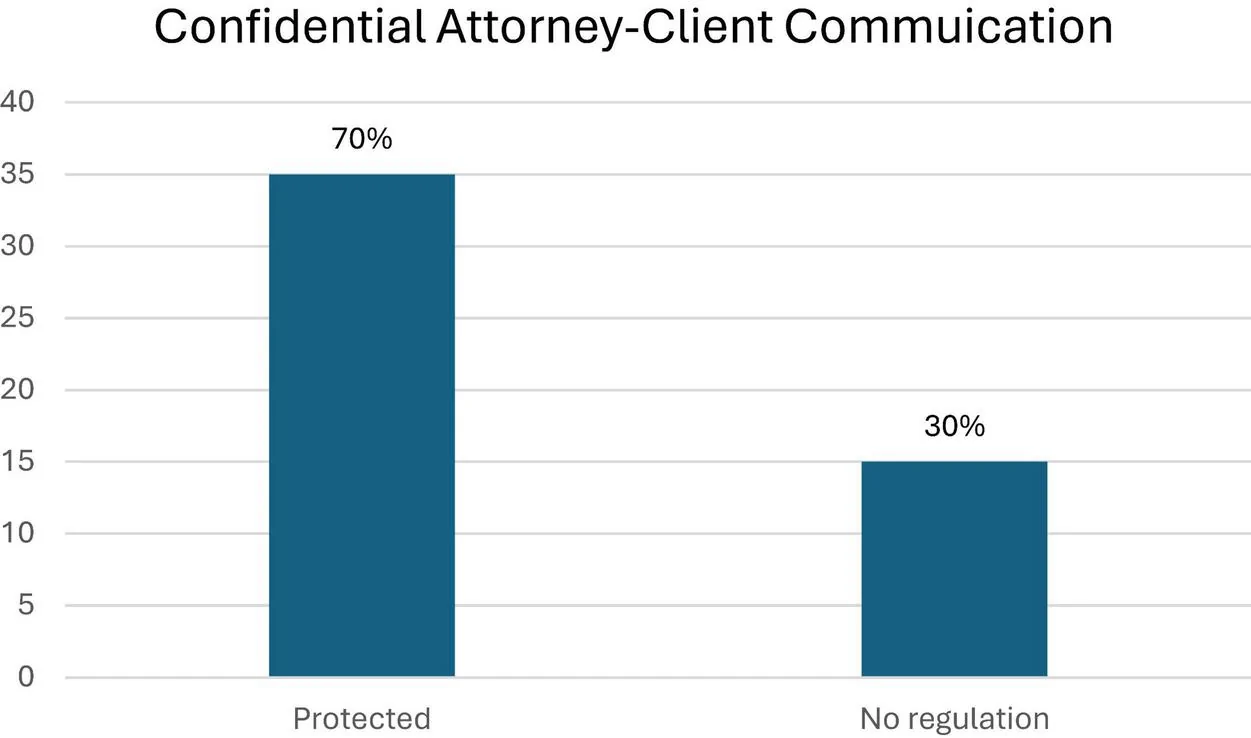
Statutes and regulations on protecting confidential attorney-client communication in remote criminal hearings.

The protection offered in several states is notably limited. In Nevada and Wyoming, defense attorneys are advised to privately communicate with their clients by requesting a recess or using text messages or emails [Nev. Rules of Virtual Advocacy R. 3 (2024); Wyo. R. Crim. P. 43.1 (2022)]. These approaches are inadequate, particularly for detained defendants who often lack access to the necessary technology. North Dakota requires parties to coordinate with the court to set up private communication channels, while Oklahoma and South Carolina explicitly state that courts are not responsible for providing methods of private communication [N.D. Sup. Ct. Admin. R. 52 (2024); Okla. Dist. Ct. R. 34 (2018); S.C. Sup. Ct. Order No. 2021-09-21-01 (2021)]. Fourteen states and the federal court system have not adopted rules that address confidential attorney-client communication during remote criminal hearings.

Another method of protecting defendants’ right to effective attorney representation is to require defendants and defense counsel to appear from the same physical remote location (Clarke and Smith, 2021; Turner, 2022). Colorado, Minnesota, Mississippi, Montana, New Mexico, New York, and North Dakota are the seven states (14%) that provide this right, and most of them allow defendants to waive the requirement. For example, in North Dakota, “an attorney for a defendant must be present at the site where the defendant is located unless the attorney’s participation by reliable electronic means from another location is approved by the court with the consent of the defendant” [N.D. Sup. Ct. Admin. R. 52 (2024)]. Michigan, Ohio, and Wisconsin adopt a different approach that allows defense attorneys to request to appear remotely with the defendant from the same location. According to Ohio Rules of Criminal Procedure, Rule 43 (2023), “counsel shall be permitted to appear with defendant at the remote location if requested.”

### 4.2 Improper influence over remote witnesses

As shown in Table 2 and Figure 2, among the 31 states that allow witnesses and/or sworn police officers to participate remotely, only eight states (25.8%) have adopted regulations to mitigate the risk of improper pressure or influence over remote witnesses. These states employ safeguards such as implementing measures to prevent witnesses from communicating with others or accessing undisclosed material, requiring witnesses to turn off electronic devices not used for the hearing, regulating locations from which witnesses testify, and verifying that witnesses are alone. Some states like Illinois use broad language that requires judges to confirm appropriate safeguards against improper influences are available and operational before permitting remote testimony [Ill. Sup. Ct. R. 45 (2023)]. Others adopt specific safeguards such as ordering witnesses to share their surroundings or any materials used during the testimony [Md. Rule 21-301 (2025); Va. Code Ann. §19.2-188.4 (2022)].

**TABLE 2:** Statutes and regulations on improper influence over remote witnesses.

Mitigate improper influence over remote witnesses (*N* = 8; 25.8%)	No regulation (*N* = 23; 74.2%)
Alaska Illinois Maine Maryland Michigan Nevada Vermont Virginia	Alabama California Florida Georgia Hawaii Idaho Iowa Kansas Louisiana Minnesota New Mexico New York North Carolina North Dakota Ohio Oklahoma Oregon South Dakota Tennessee Texas West Virginia Wisconsin Wyoming

**FIGURE 2:**
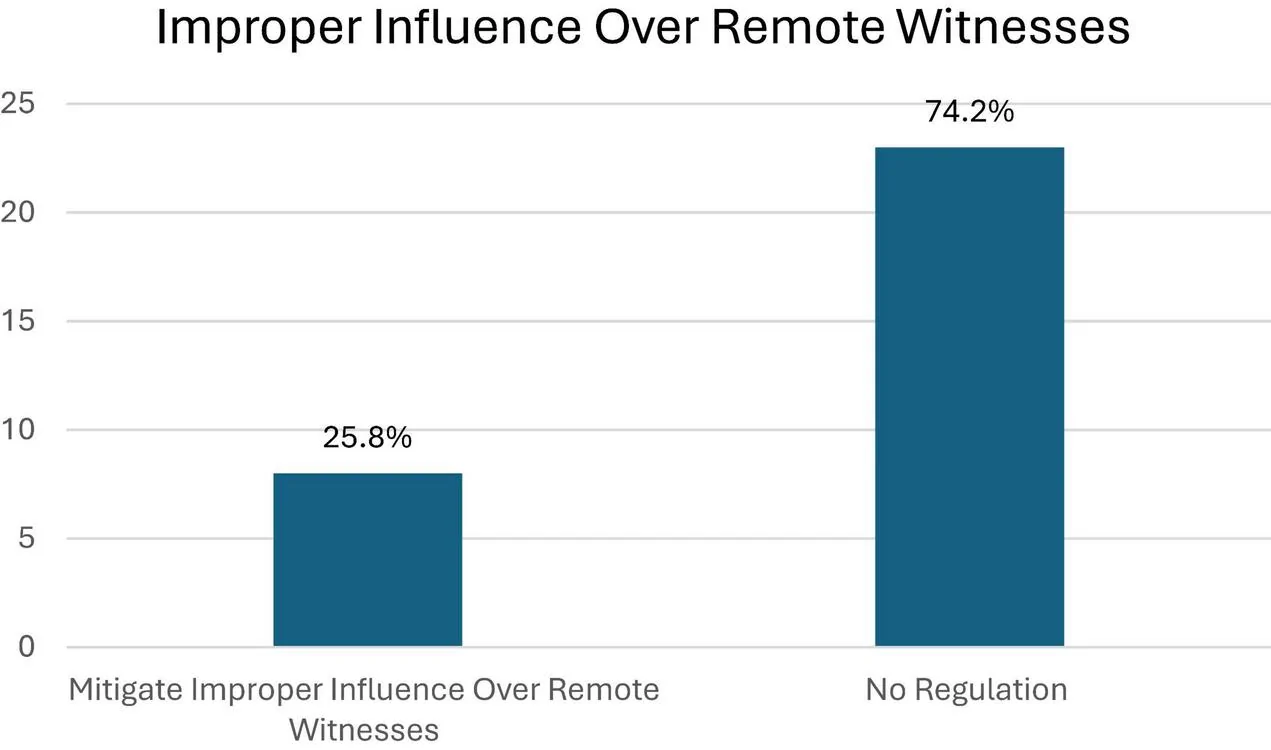
Statutes and regulations on improper influence over remote witnesses.

### 4.3 Audio and/or video quality

Ensuring high-quality audio, video, and connectivity is essential for the integrity of remote proceedings. As shown in Table 3 and Figure 3, thirty-two states (64%) require, at a minimum, that all participants in VTC hearings can see, hear, and communicate with one another simultaneously. For example, according to Mississippi Rules of Criminal Procedure, Rule 1.8 (2017), VTC equipment used for remote hearings “shall at a minimum operate so as to enable the court and all parties to view and converse with each other.” Among them, nine states further require that the VTC equipment sufficiently allows all participants to observe each other’s demeanor, facial expressions, or activities occurring in the courtroom. For example, one of the technical and operational standards for Wisconsin’s VTC hearings is that “video and sound quality shall be adequate to allow participants to observe the demeanor and non-verbal communications of other participants and to clearly hear what is taking place in the courtroom to the same extent as if they were present in the courtroom” [Wis. Stat. Ann. §885.54 (2008)]. To prevent technical challenges, Arizona also asks court staff to perform daily system checks and verify the audio function after any loss of power or system shutdown [Ariz. Code Jud. Admin. §5-208 (2023)]. However, 16 states, the District of Columbia, and the federal court system have not adopted rules on the quality of VTC connections.

**TABLE 3:** Statutes and regulations on video and/or audio communication quality.^1^

See, hear, and communicate simultaneously (*N* = 32; 64%)	Observe demeanor (*N* = 9; 18%)	No regulation (*N* = 18; 36%)
Alabama Alaska Arizona Arkansas California Colorado Connecticut Florida Georgia Idaho Maryland Michigan Mississippi Montana Nebraska Nevada New Mexico New York North Carolina Ohio Oklahoma Oregon Pennsylvania South Dakota Tennessee Texas Vermont Virginia Washington West Virginia Wisconsin Wyoming	Arizona Arkansas Georgia Maryland Michigan North Carolina Oklahoma Tennessee Wisconsin	Delaware District of Columbia Federal Hawaii Illinois Iowa Kansas Louisiana Maine Massachusetts Minnesota Missouri New Hampshire New Jersey North Dakota Rhode Island South Carolina Utah

^1^A state may appear in multiple columns because it provides multiple types of protection.

**FIGURE 3:**
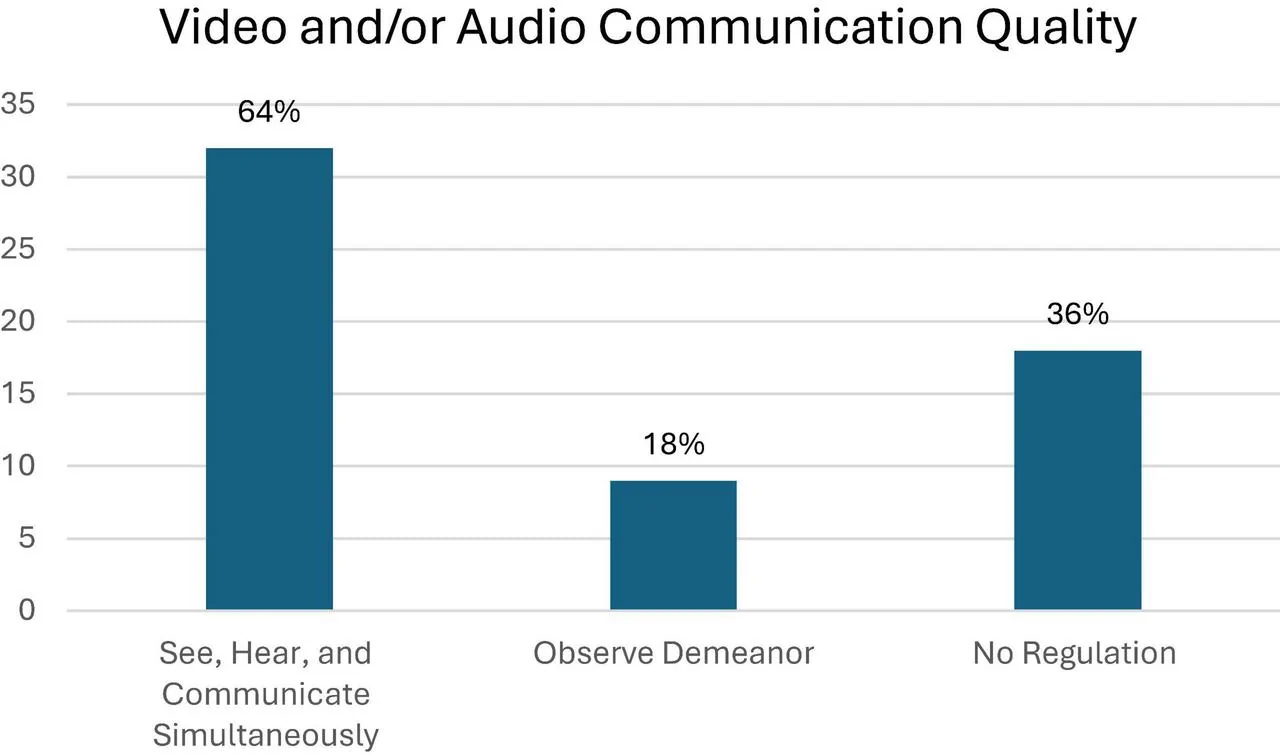
Statutes and regulations on video and/or audio communication quality. A state may appear in multiple columns because it provides multiple types of protection.

### 4.4 Remote document and exhibit management

Another challenge of remote criminal hearings is document and exhibit management. To facilitate the process, 19 states allow participants and/or the court to transfer documents and exhibits electronically or share them on the screen. For example, in Oklahoma, “any pleading, other document, or exhibit used in a proceeding conducted by videoconferencing may be transmitted between the court’s location and any remote site by electronic means, including, but not limited to facsimile, scan, or email” (Okla. Dist. Ct. R. 34 (2018)). Seven states ask participants and/or the court to provide electronic and/or hard copies of documents or exhibits before the remote proceedings. For example, Connecticut Practice Book 1998, Section 44-10A (2023) states that “prior to any proceeding in which a person appears by means of an interactive audiovisual device, copies of all documents which may be offered at the proceeding shall be provided to all counsel and self-represented parties in advance of the proceeding.” However, only eight states (16%) mandate that all participants can observe or access exhibits and documents presented during remote hearings. Arkansas is one of these states. Its Rules of Criminal Procedure, Rule 8.7 (2013) states that “all participants in the proceeding must be able to see, hear, and otherwise observe any physical evidence or exhibits presented during the proceeding, either by video, facsimile, or other method.” Three other states either require that documents can be transmitted between defendants and defense attorneys, or between detained individuals and the court during remote hearings, or require that copies of court documents be provided to defendants promptly. Twenty-two states, the District of Columbia, and the federal court system (48%) have yet to address the document and exhibit management issues in remote criminal hearings. Table 4 and Figure 4 present these results.

**TABLE 4:** Statutes and regulations on document and exhibit management.^1^

May share documents electronically or on screen (*N* = 19; 38%)	Provide hard and/or electronic copies before a hearing (*N* = 7; 14%)	Must be able to observe exhibits and documents (*N* = 8; 16%)	Other (*N* = 3; 6%)	No regulation (*N* = 24; 48%)
Alabama Alaska Arkansas Colorado Delaware Florida Georgia Maine Maryland Michigan North Dakota Oklahoma Oregon South Carolina South Dakota Texas Vermont Virginia West Virginia	Connecticut Florida Maine Michigan Nevada New Mexico South Carolina	Arkansas Georgia Maryland Michigan Oklahoma South Carolina Vermont Wisconsin	Arizona Nebraska Washington	California District of Columbia Federal Hawaii Idaho Illinois Iowa Kansas Louisiana Massachusetts Minnesota Mississippi Missouri Montana New Hampshire New Jersey New York North Carolina Ohio Pennsylvania Rhode Island Tennessee Utah Wyoming

^1^A state may appear in multiple columns because it provides multiple types of protection.

**FIGURE 4:**
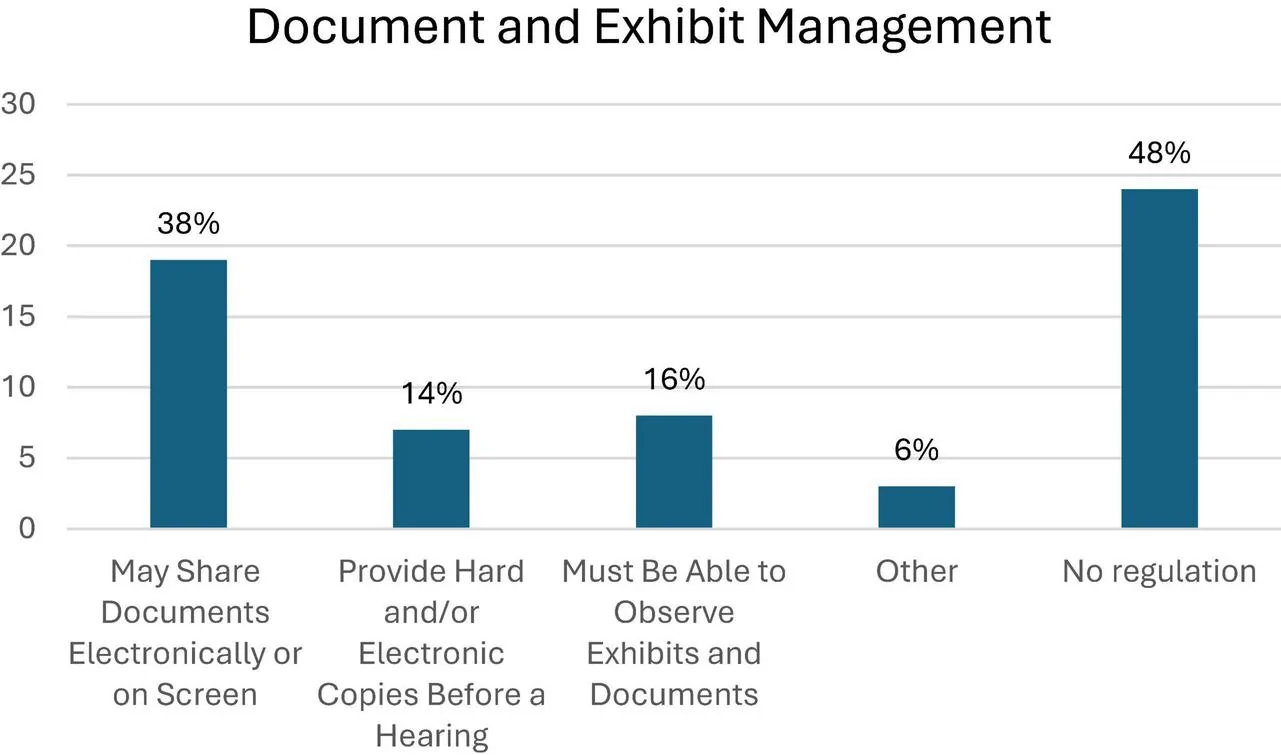
Statutes and regulations on document and exhibit management. A state may appear in multiple columns because it provides multiple types of protection.

### 4.5 Public access to remote hearings

As shown in Table 5 and Figure 5, 27 states (54%) have adopted regulations that protect public access to remote criminal hearings. For example, according to Maryland Rules, Rule 21-104 (2023), “if a proceeding that otherwise would be open to the public is conducted entirely by remote electronic means, the court shall ensure that members of the public shall have the ability to listen to the non-redactable portions of the proceeding during the course of the proceeding through remote electronic means.” Public access is provided through various methods, such as online video or audio streaming, broadcasting proceedings at a publicly accessible location, and allowing public members to join a video conference without the ability to participate.

**TABLE 5:** Statutes and regulations on public access to remote hearings.

Protected (*N* = 27; 54%)	No regulation (*N* = 23; 46%)
Alabama Alaska Arizona Colorado Delaware Florida Georgia Illinois Iowa Kansas Maine Maryland Massachusetts Michigan Minnesota Nebraska Nevada New Mexico New York North Carolina Oklahoma Oregon South Carolina Tennessee Vermont Washington Wisconsin	Arkansas California Connecticut District of Columbia Federal Hawaii Idaho Louisiana Mississippi Missouri Montana New Hampshire New Jersey North Dakota Ohio Pennsylvania Rhode Island South Dakota Texas Utah Virginia West Virginia Wyoming

**FIGURE 5:**
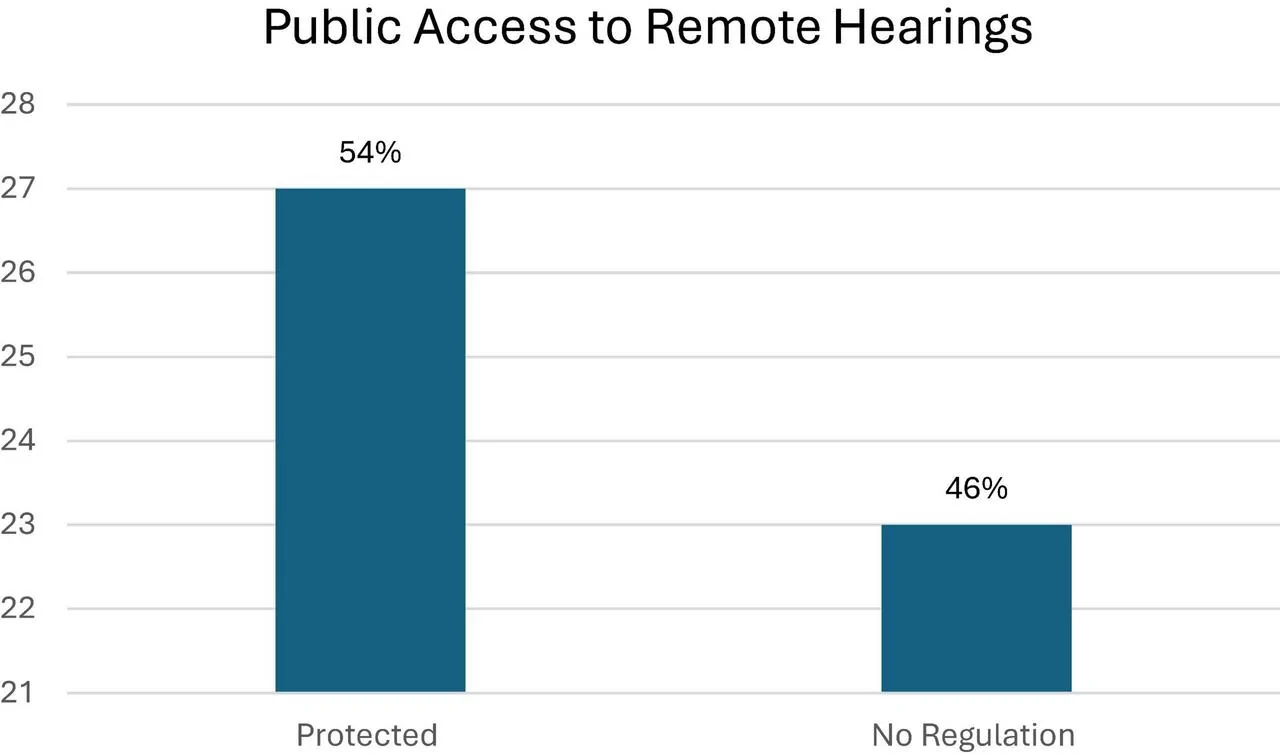
Statutes and regulations on public access to remote hearings.

## 5 Discussions and conclusion

This study examines the extent to which state and federal laws incorporate procedural safeguards in remote criminal proceedings and mitigate challenges posed by video teleconferencing technology. It focuses on five concerns, including (1) barriers to defendants’ right to effective assistance of counsel, (2) improper influence over remote witnesses, (3) poor VTC audio and/or video quality, (4) ineffective document and exhibit management, and (5) public access to remote hearings. The findings are troubling: across the nation, these concerns are addressed in at most 70% of the applicable states and at worst in fewer than 30%. Moreover, as shown in Appendix 1, Alaska, Arizona, Colorado, Maryland, Michigan, Nebraska, Nevada, Vermont, and Washington are the only nine states that have adopted rules in all five areas. Meanwhile, six states and the federal government have not implemented any regulations addressing the foregoing concerns.

Preventing improper influence over remote witnesses is the least recognized safeguard, codified in fewer than 30% of the states that authorize remote testimony. One possible explanation for this nationwide gap is that there is currently limited literature on best practices for preventing witness coaching and coercion. Although legal scholars have recommended methods such as requiring witnesses to share their surroundings or to appear from court-approved locations (Benninger et al., 2021; Lederer, 2021; Prosecutors’ Center for Excellence, 2024), these approaches have not been comprehensively evaluated. Legislators can nevertheless begin by requiring judges to verify on the record that a witness is not assisted by an authorized person or document. Such a requirement can raise judicial awareness of the risk of improper influence, encourage judges to take preventive measures, and may deter misconduct.

Meanwhile, slightly more than half of the states have implemented rules governing participants’ access to documents and exhibits during remote hearings, with only eight expressly mandating that all parties be able to observe or access these case materials. Without clear guidance, participants may resort to improper methods or find the process confusing (Bannon and Keith, 2021). Inadequate access to hearing documents and case materials may also raise serious constitutional concerns (Jurva, 2021). Moreover, inefficient document management may negate the efficiency gains that motivate courts to use VTC (Clarke and Smith, 2021; Yamagata and Fox, 2017). As recommended by European Commission for the Efficiency of Justice [CEPEJ] (2021), courts should provide instructions on how participants present documents and other case materials during remote hearings and make practical arrangements to ensure all participants can see and/or hear the materials presented.

Only just over half of the states have regulations that protect public access to remote criminal hearings, leaving gaps in court transparency. Court administrators should establish clear rules governing public access to remote hearings that balance transparency with protecting privacy and information security (European Commission for the Efficiency of Justice [CEPEJ], 2021; National Center for State Courts [NCSC], 2025). Legal scholars and court organizations have recommended methods such as live-streaming remote hearings at public viewing rooms, using a delayed stream, or requiring public observers to complete a registration form (Boulanger-Bonnelly, 2022; National Center for State Courts [NCSC], 2025).

Even when most states have responded to a procedural challenge, their protective measures remain limited in scope. Although 70% of states have rules on confidential attorney-client communication for remote hearings, only a few states inform defendants and counsel on how to exercise this right or protect it before a hearing begins. These gaps undermine defense attorneys’ ability to gather information and advocate effectively, and make it more difficult for defendants to seek timely assistance, both of which affect the quality of counsel that defendants receive (Diamond et al., 2010; Turner, 2022). In addition, although 64% of states have adopted rules governing VTC quality, most impose only a minimal requirement that all participants can see, hear, and communicate with each other. Only a minority of states require that the video and audio quality be sufficient for participants to observe each other’s demeanor and non-verbal cues, which are critical for assessing participants, particularly witnesses who appear remotely (Bandes and Feigenson, 2020; Feigenson, 2023). Without these more robust measures, the laws in most states offer only superficial protection that cannot meaningfully mitigate challenges posed by remote hearings.

Such nationwide deficiencies raise concerns about whether legislators and court administrators have adequately evaluated the differences between in-person and remote hearings when authorizing remote criminal proceedings. Without uniform and robust standards for the protection of rights, courts are relying on individual judges’ discretion to address procedural justice challenges. Yet, an observation of remote plea hearings showed that few judges offered procedural safeguards beyond those expressly required by law (Turner, 2022). Moreover, not all judges received adequate training or guidance on preserving procedural justice in remote hearings (Clark, 2021). These concerns suggest that gaps in current state and federal legal codes may undermine courts’ ability to make remote hearings comparable to in-person proceedings.

These challenges are not unique to the United States. The Council of Europe, through its European Commission for the Efficiency of Justice, has also recognized the need for stronger procedural protections and recommended that its member States establish a legal framework that protects parties’ rights to be heard, to actively participate, and to exercise their right to a defense (European Commission for the Efficiency of Justice [CEPEJ], 2021). Specifically, European Commission for the Efficiency of Justice [CEPEJ] (2021) recommended adopting statutory safeguards to ensure defendants have access to secure, surveillance-free communication channels with their counsel; prevent pressure or influence on remote witnesses; continuously monitor the hearing’s audio-visual quality; preserve the public nature of remote hearings; and ensure that all participants can view case materials presented during remote hearings. Similarly, the Netherlands has adopted a Videoconference Decree that requires the videoconferencing systems to guarantee confidential communication, truthfully represent courtroom events, and facilitate remote file exchange (Ballin and Castelijn, 2024). These approaches provide concrete models for United States policymakers and court administrators to develop comprehensive statutory protections that move beyond individual judicial discretion and establish uniform standards for remote proceedings.

Proactively regulating the use of VTC can encourage policymakers to anticipate challenges likely to arise in remote criminal courts and establish clear and uniform standards to address them effectively (European Commission for the Efficiency of Justice [CEPEJ], 2021). In doing so, such regulations also enhance the courts’ perceived fairness and transparency, which can strengthen defendants’ perceptions of courts as legitimate authorities and increase their willingness to accept court rules and decisions, even when the outcomes are unfavorable (Tyler, 2003). In addition, the clear articulation of procedural protections in legal codes helps defendants make informed decisions about consenting to VTC, particularly in jurisdictions where courts are required to obtain defendants’ consent to use VTC. Establishing strong statutory protections helps courts take advantage of the efficiency and accessibility benefits of remote hearings while reducing the risk that these benefits come at the expense of justice.

It is important to recognize that courtroom technologies and regulations have been and will likely continue to change. The study is only a snapshot of procedural safeguards currently codified in statewide and federal statutes and regulations. It also does not necessarily reflect individual courtroom practices, nor does it include local or internal rules. In addition, most of the current literature on remote criminal courts is theoretical or based on studies conducted before or during the early COVID-19 era. As technologies evolve and both courts and public members become more familiar and comfortable with VTC, the implications of conducting remote hearings may change substantially. This study’s findings are time-sensitive and should be updated periodically to reflect advances in technology, legislation, and further legal and empirical analyses. Additional empirical research, such as case studies of remote criminal proceedings and studies examining how VTC affects hearing processes and outcomes, is needed to better understand its effect and identify effective ways to address potential challenges. Furthermore, this study focuses on general procedural safeguards across different types of remote criminal proceedings. As a result, it may not capture differences in the protections required for evidentiary and non-evidentiary hearings or between fully remote and hybrid proceedings. Finally, the analysis was conducted by a single researcher, which may introduce subjectivity in the interpretation and classification of legal provisions.

Establishing statutory and regulatory safeguards is only one of the many essential elements to address all procedural justice concerns. Other legal actors could also play critical roles in promoting fair remote criminal hearings. Even with robust statutory safeguards and confidential communication systems, defense attorneys may still struggle to communicate with clients in immediate and subtle ways (Clarke and Smith, 2021). How participants are portrayed by VTC can be affected by small factors such as camera angles, camera locations, or lighting (Lederer, 2021), which are usually more appropriately addressed on a case-by-case basis instead of through statewide regulations. Remote defendants may also feel disengaged and have trouble understanding the court (Clarke and Smith, 2021; McKay, 2018; Nir and Musial, 2022). Judges can exercise their discretion to address concerns that legal protections cannot remedy. For instance, they can start a remote hearing by explaining how it will unfold, periodically check whether defendants understand the proceeding or wish to consult with counsel privately, and monitor the VTC quality (Rossner and Tait, 2024). Courts can implement mandatory administrative guidelines to promote consistency in how judges implement protective measures. They can also provide judges with comprehensive training on the challenges associated with remote hearings and ways to mitigate the risks.

In conclusion, the U.S. judicial system has not implemented adequate statutory procedural safeguards to address challenges posed by remote criminal proceedings. Without these protections, remote proceedings may struggle to achieve the same standards of justice as in-person proceedings. Legislators and court administrators should carefully evaluate the risks and challenges presented by VTC and establish robust, evidence-based procedural safeguards.

## Data Availability

The original contributions presented in the study are included in the article/supplementary material, further inquiries can be directed to the corresponding author.

## Appendix 1

**TABLE A1:** A procedural safeguards in the 50 states, District of Columbia, and Federal Courts.

States	Confidential attorney-client communication	Improper influence over witnesses	VTC quality	Exhibit and document management	Public access
Alabama	Y	N	Y	Y	Y
Alaska	Y	Y	Y	Y	Y
Arizona	Y	NA	Y	Y	Y
Arkansas	Y	NA	Y	Y	N
California	Y	N	Y	N	N
Colorado	Y	NA	Y	Y	Y
Connecticut	Y	NA	Y	Y	N
Delaware	N	NA	N	Y	Y
Florida	N	N	Y	Y	Y
Georgia	Y	N	Y	Y	Y
Hawaii	N	N	N	N	N
Idaho	Y	N	Y	N	N
Illinois	Y	Y	N	N	Y
Iowa	N	N	N	N	Y
Kansas	Y	N	N	N	Y
Louisiana	Y	N	N	N	N
Maine	N	Y	N	Y	Y
Maryland	Y	Y	Y	Y	Y
Massachusetts	N	NA	N	N	Y
Michigan	Y	Y	Y	Y	Y
Minnesota	Y	N	N	N	Y
Mississippi	Y	NA	Y	N	N
Missouri	N	NA	N	N	N
Montana	Y	NA	Y	N	N
Nebraska	Y	NA	Y	Y	Y
Nevada	Y	Y	Y	Y	Y
New Hampshire	N	NA	N	N	N
New Jersey	N	NA	N	N	N
New Mexico	Y	N	Y	Y	Y
New York	Y	N	Y	N	Y
North Carolina	Y	N	Y	N	Y
North Dakota	Y	N	N	Y	N
Ohio	Y	N	Y	N	N
Oklahoma	Y	N	Y	Y	Y
Oregon	Y	N	Y	Y	Y
Pennsylvania	Y	NA	Y	N	N
Rhode Island	N	NA	N	N	N
South Carolina	Y	NA	N	Y	Y
South Dakota	Y	N	Y	Y	N
Tennessee	N	N	Y	N	Y
Texas	Y	N	Y	Y	N
Utah	N	NA	N	N	N
Vermont	Y	Y	Y	Y	Y
Virginia	N	Y	Y	Y	N
Washington	Y	NA	Y	Y	Y
West Virginia	N	N	Y	Y	N
Wisconsin	Y	N	Y	Y	Y
Wyoming	Y	N	Y	N	N
District of Columbia	Y	NA	N	N	N
Federal	N	NA	N	N	N

^1^Indiana and Kentucky are excluded from the analysis because they did not have regulations that specifically permit defendants to appear at a criminal hearing remotely at the time of data collection.
